# *Pseudorogneria libanotica* Intraspecific Genetic Polymorphism Revealed by Fluorescence In Situ Hybridization with Newly Identified Tandem Repeats and Wheat Single-Copy Gene Probes

**DOI:** 10.3390/ijms232314818

**Published:** 2022-11-26

**Authors:** Dandan Wu, Namei Yang, Qian Xiang, Mingkun Zhu, Zhongyan Fang, Wen Zheng, Jiale Lu, Lina Sha, Xing Fan, Yiran Cheng, Yi Wang, Houyang Kang, Haiqin Zhang, Yonghong Zhou

**Affiliations:** 1 State Key Laboratory of Crop Genetic Exploration and Utilization in Southwest China, Sichuan Agricultural University, Chengdu 611130, China; 2 Triticeae Research Institute, Sichuan Agricultural University, Chengdu 611130, China; 3 College of Grassland Science and Technology, Sichuan Agricultural University, Chengdu 611130, China

**Keywords:** fluorescence in situ hybridization, homoeologous chromosome, tandem repeat, cytogenetic karyotype, genetic polymorphism, *Pseudoroegneria libanotica*

## Abstract

The genus *Pseudoroegneria* (Nevski) Löve (Triticeae, Poaceae) with its genome abbreviated ‘St’ accounts for more than 60% of perennial Triticeae species. The diploid species *Psudoroegneria libanotica* (2n = 14) contains the most ancient St genome. Therefore, investigating its chromosomes could provide some fundamental information required for subsequent studies of St genome evolution. Here, 24 wheat cDNA probes covering seven chromosome groups were mapped in *P. libanotica* to distinguish homoelogous chromosomes, and newly identified tandem repeats were performed to differentiate seven chromosome pairs. Using these probes, we investigated intraspecific population chromosomal polymorphism of *P. libanotica*. We found that (i) a duplicated fragment of the 5St long arm was inserted into the short arm of 2St; (ii) asymmetrical fluorescence in situ hybridization (FISH) hybridization signals among 2St, 5St, and 7St homologous chromosome pairs; and (iii) intraspecific population of polymorphism in *P. libanotica.* These observations established the integrated molecular karyotype of *P. libanotica.* Moreover, we suggested heterozygosity due to outcrossing habit and adaptation to the local climate of *P. libanotica.* Specifically, the generated STlib_96 and STlib_98 repeats showed no cross-hybridization signals with wheat chromosomes, suggesting that they are valuable for identifying alien chromosomes or introgressed fragments of wild relatives in wheat.

## 1. Introduction

The tribe Triticeae (Poaceae) represents vital germplasm, including economically important annual crops (e.g., wheat, rye, triticale, and barley) as well as crucial perennial forage grasses (e.g., *Roegneria* C. Koch, *Agropyron* Gaertn., *Leymus* Hochst, *Pseudoroegneria* (Nevski) Á. Löve). The genus *Pseudoroegneria* with the St genome accounts for eight alloploid genera formations, including *Trichopyrum* Á. Löve, *Elymus* L., *Roegneria* C. Koch, *Douglasdeweya* C. Yen, J. L. Yang et B. R. Baum, *Campeiostachys* Drobov, *Kengyilia* C. Yen et J. L. Yang, *Psammopyrum* Á. Löve, *Anthosachne* Steudel, and *Pascopyrum* Á. Löve [1,2,3]. Hence, characterization of the St genome is critical for a better understanding of the Triticeae species evolution. The *Pseudoroegneria* derived from 14.4–14.7 million years (Myr), which indicates that it is more ancient than the *Triticum/Aegilops* group (8.0–8.3 Myr) [4]. It consists of six diploid (2n = 2x = 14, StSt) and nine autotetraploid species (2n = 4x = 28, StStStSt). Species that belong to the *Pseudoroegneria* species are predominately cool-season grasses, and are drought- and salt-tolerant, making them highly desirable for plant breeders.

Interspecific hybridizations of the diploid *Pseudoroegneria* [5] and molecular studies [6,7] indicate that more than one form of the St genome is present within the *Pseudoroegneria* genus, and they are polyphyletic originated. *P. libanotica* (Hack.) D. R. Dewey from Western Asia is placed at the base of the phylogenetic tree, implying that *Pseudoroegneria* species distributed in Western Asia are likely to be more ancient than those found in species from Eastern Europe (*Pseudoroegneria strigosa* M. Bieb.), East Asia (*Pseudoroegneria* [Czern. ex Nevski] Á. Löve), and North America (*Pseudoroegneria spicata* [Pursh] Á. Löve) [8,9]. Thus, *P. libanotica* might possess a more ancient St genome, and investigating its chromosomes could set the stage for subsequent studies of St genome evolution. Few studies have focused on the intraspecific nucleotide diversity that exists within *Pseudorogneria* species [8,10]. However, they could not visualize that genetic diversity because of limited cytogenetic markers and unclassified homoeologous chromosome groups.

In general, genic sequences and their order are generally relatively conserved in grass genomes [11]. Bacterial artificial chromosome (BAC) clones are used as fluorescence in situ hybridization (FISH) probes for genic regions in species with small genomes and low content of repetitive elements [12,13,14], such as *Brachypodium distachyon* (L.) P. Beauvois [12]. In plant species with larger genomes, cDNA sequences are applied as FISH probes as they allowed chromosome identification, chromosome rearrangements investigation, and study of the homoeologous relationship between related Triticeae species, such as *Hordeum vulgare* L. [12,15], *Triticum aestivum* L. and its wild relatives (i.e., *Aegilops caudata* L., *Aegilops comosa* Sibth. Et Sm., *Aegilops markgrafii* (Greuter) Hammer, and *Aegilops mbellulate* Zhuk.) [16,17,18,19], as well as perennial relative *Agronpyron cristatum* (Linn.) Gaertn. [20]. The expression sequence tag-simple sequence repeats (EST-SSRs) and genotype sequencing revealed that St genome species and the three subgenomes of common wheat presented high collinearity [21]. The homoeology between *P. libanotica* and other Triticeae species’ chromosomes has not been investigated in detail. Hence, developing a single-gene FISH map for the St genome could help with determination of the homoeologous chromosome group and uncovering the inter/intraspecific diversity of *Pseudorogneria* species.

Repetitive DNA sequences evolve rapidly and can be used to distinguish single chromosomes, identify repeat sequences distribution on chromosomes, and detect gene insertion sites. Numerous repeats have been applied to identify homoeologous chromosomes in Triticeae species. This can eliminate the karyotype inconsistency caused by different treatment methods and can act as a powerful tool for studying species variation and kindship [22,23,24]. Only a few cytogenetic markers have been employed to determine the whole St sub-genome in allopolyploids [25,26], however, those cytogenetic markers could not distinguish seven homoeologous chromosome groups. An attempt was made to differentiate each chromosome pair in StY genome species [27], whereas the presence and copy number of repetitive elements vary among Triticeae species. Thus, developing more specific repetitive cytogenetic markers for the St genome homoeology would be advantageous. Similarity-based clustering of Illumina reads implemented in the REPEATEXPLORER pipeline was used to identify repeat probes in species without reference genomes [28,29,30], which is promising for identifying cytogenetical markers in *P. libanotica*. 

In this study, we determined the karyotype of *P. libanotica* and analyzed the intraspecific population chromosomal polymorphism by using a set of wheat cDNA sequences and newly identified repeats. Our results provide a cytogenetic perspective on the genetic diversity of perennial Triticeae species. The generated STlib_96 and STlib_98 repeats have the potential for application in determining the alien St chromosome or introgressed segments in common wheat.

## 2. Results

### 2.1. Identification of Homoeologous Chromosomes in P. libanotica Based on Wheat Single-Copy Genes

**Figure 1:**
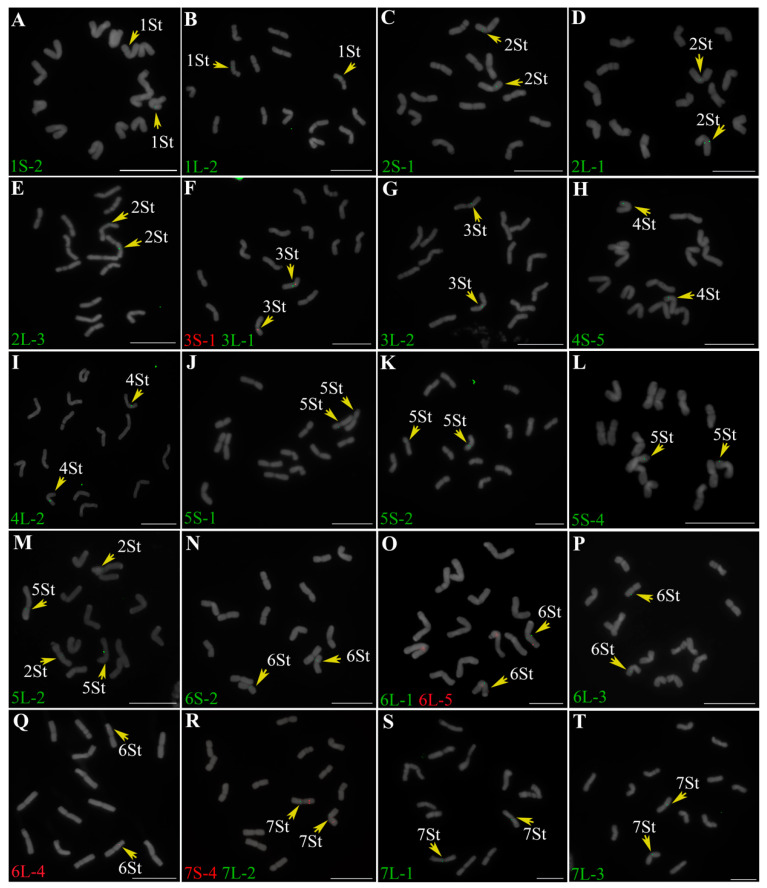
Common wheat single-copy gene FISH probes to distinguish seven homoeologous chromosome groups of *P. libanotica*. (**A**,**B**) 1S-2 and 1L-2 in green were identified the 1St chromosome pair. (**C**–**E**) 2S-1, 2L-1, and 2L-3 in green were displayed signals on the 2St chromosome pairs. (**F**,**G**) 3S-1 in red, 3L-1 and 3L-2 in green were presented on the 3St chromosome pair. (**H**,**I**) 4S-5 and 4L-2 in green were visualized on the 4St chromosomes. (**J**–**M**) 5S-1, 5S-2, 5S-4 in green were localized on the short arm of 5St, 5L-2 was hybridized with 2St and 5St chromosomes. (**N**–**Q**) 6S-2, 6L-1, and 6L-3 in green, 6L-4 and 6L-5 in red were distributed on the 6St chromosomes. (**R**–**T**) 7S-4 in red, 7L-1, 7L-2, and 7L-3 in green were observed on the 7St chromosomes. Probes were labeled green and red fluorescein. The homoeologous chromosome group was marked with yellow arrows. Scale bar equals 10 μm.

**Figure 2:**
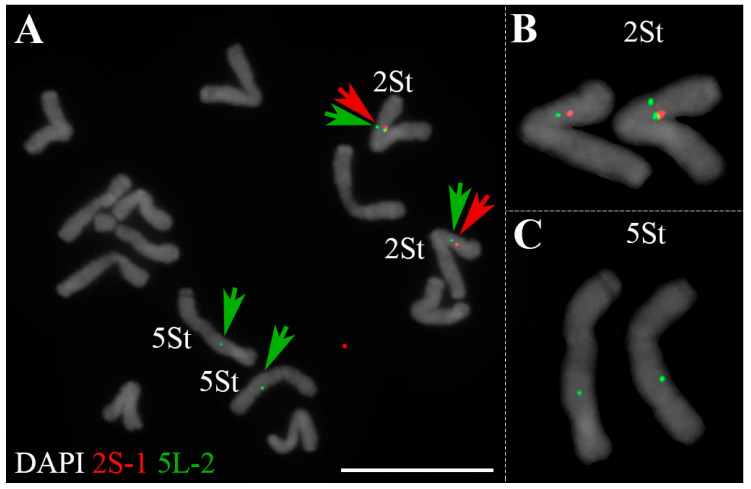
Single-copy gene duplication in the chromosome of the *P. libanotica*. (**A**) Mitotic chromosomes of *P. libanotica* with single-copy cDNA of 5L-2 (in green) and 2S-1 (in red). (**B**) cDNA FISH probes 5L-2 and 2S-1 located on the 2St_S chromosome. (**C**) cDNA FISH probes 5L-2 labeled the 5St chromosome. 2St and 5St were marked with red and green arrows, respectively. Scale bar equals 10 μm.

We applied 24 single-copy gene FISH probes specific for the seven homoeologous groups in wheat to recognize chromosome pairs of *P. libanotica* PI 228392. Most probes were cross-hybridized with *P. libanotica* as expected (Figure 1). Probe 5L-2 was supposed to be located on the long arm of 5St (5St_L), exhibiting four signals in two chromosome pairs (Figure 1I). We performed double-colored cDNA-FISH by using 2S-1 and 5L-2 probes to confirm the location of the extra 5L-2 copy (Figure 2). The additional 5L-2 signal was distributed on the short arm of 2St (2St_S) near the centromere. Moreover, we found that the duplication was conserved within different accessions of *P. libanotica* (Figure S1).

Based on relative cDNA-FISH sites position, we grouped the homoeologous chromosomes *P. libanotica* PI 228392 (Table 1) and characterized the karyotype information in Table 2. In *P. libanotica*, the chromosome sizes ranged from 4.35 to 5.59 μm. Based on the centromere position and arm ratio, we classified those chromosomes into two different types. Among them, five chromosomes were metacentric (2St, 3St, 4St, 6St, and 7St), with the remaining being submetacentric and having a secondary constriction on the short arm (1St and 5St). The relative chromosome length ranged from 16.13% for the largest chromosome (2St) to 12.20% for the smallest chromosome (4St) (Table 2).

### 2.2. Characterisation of the P. libanotica Repeat Composition and Identification of Satellite Repeats 

To explore the repeat composition of *P. libanotica*, we utilized paired-end Illumina sequencing and obtained approximately 2 Gbp (corresponding to ~0.5 × coverage) data; a total of 16,843,012 reads were applied for similarity-based clustering by using the REPEATEXPLORER pipeline [30]. Ty3/gypsy elements (14.50%) were found to be more abundant than Ty1/copia elements (8.14%) (Table 3). The Ty3/gypsy superfamily was mostly represented by the elements from the Tekey (5.87%) and Athila (5.34%) lineages. For Ty1/copia elements, Angela (5.68%) was most common, followed by the Sire lineage (2.29%). Other repeat clusters, such as EnSpm_CACTA and satellite DNA, accounted for 3.38% and 2.66% of the genome, respectively. 

**Figure 3:**
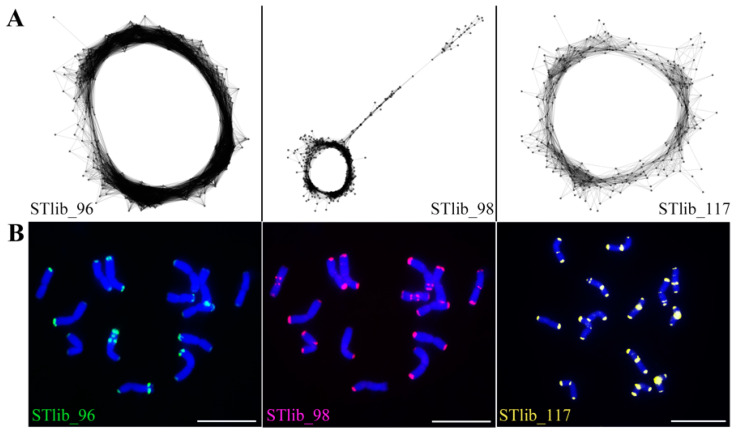
Identification and characterization of *P. libanotica* enriched sequences. (**A**) Graph representation of repeat clusters read similarities of *P. libanotica* in silico. Nodes are placed according to sequence similarity, where similar sequences are close together and connected with edges (gray lines). The circular or globular structure of graphs is typical for repeats with the tandem arrangement in the genome. (**B**) Chromosome distribution of tandem repeats (STlib_96 in green, STlib_98 in magenta, and STlib_117 in yellow) in *P. libanotica*, respectively.

Three highly confident repeat clusters were developed (named STlib_96, STlib_98, and STlib_117, Figure 3A) based on analyzing the whole genome paired-end Illumina reads of *P. libanotica* PI 228392. STlib_96 (528 bp in length, with a genome proportion of 0.110%) was the satellite repeat that exhibited no similarity with sequences of other species in the NCBI database. STlib_98 (503 bp in length, with a genome proportion of 0.089%) was classified as an EnSpm/CACTA element and shared 89.15% similarity with the satellite repeat of *Ae. crassa* Bioss. (GenBank ID: ON872676). STlib_117 (352 bp in length, with a genome proportion of 0.059%) was a satellite repeat and shared 84.5% similarity with the subregion of the satellite repeat CentT550 in *T. aestivum* (GenBank ID: MN161206). 

To detect the species specificity of the repeat clusters, we conducted FISH in common wheat by combining 45S rDNA and the newly identified satellite DNA probes (Figure S2). STlib_96 and STlib_98 displayed no cross-hybridization with wheat (Figure S2A–H), whereas STlib_117 was present in seven chromosome pairs (Figure S2I–L). Thus, STlib_96 and STlib_98 could help to identify *P. libanotica* derived chromosomes and chromatin in the background of common wheat after cross-genus hybridization.

**Figure 4:**
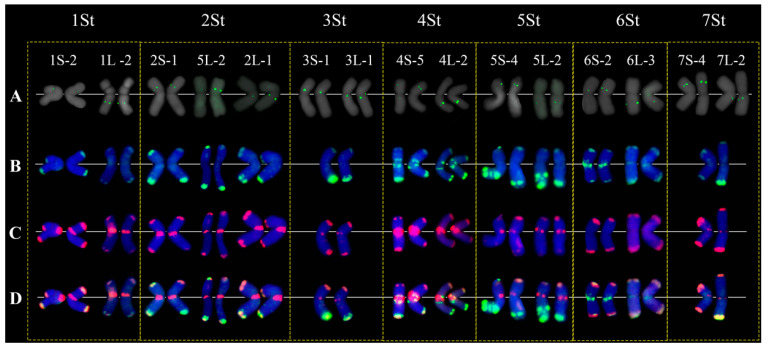
The distribution of STlib_96 + STlib_98 (green) and STlib_117 (red) after single-gene probes in *P. libanotica* PI 228392. (**A**–**D**) Cytogenetic karyotype of *P. libanotica* with 14 single-gene probes and developed repetitive sequences. (**A**) Single-gene probe in green. (**B**) Repetitive sequence STlib_96 + Stlib_98 (green). (**C**) Repetitive cluster STlib_117 (red). S and L letters refer to short and long arms, respectively. (**D**) Merged repetitive clusters from STlib_96 + Stlib_98 (green) and STlib_117 (red).

**Figure 5:**
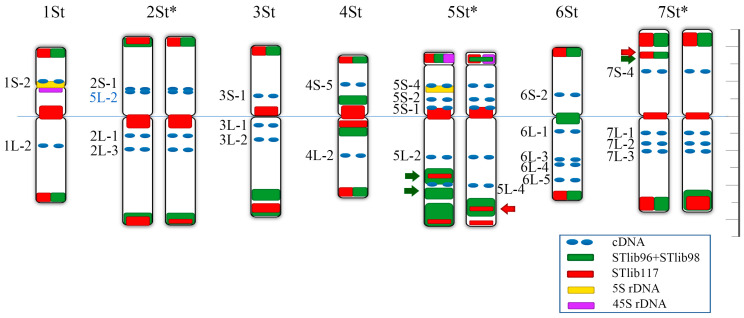
Idiogram of *P. libnotica*. Asterisks on 2St, 5St, and 7St indicate heterozygous between homologous chromosomes. Green and red arrows present repeat duplication compared with homologous chromosomes. The name of cDNA probes that hybridized to a non-homoeologous chromosome is highlighted in blue. The color scheme (bottom right) shows the color of each probe as represented in this idiogram. The scale on the right side consists of 2 units, and each unit represents 0.5 μm.

We performed FISH in *P. libanotica* using STlib_96, STlib_98, and STlib_117, and found that three probes can differentiate seven St-chromosome pairs based on the signal distribution pattern (Figure 3B). After conducting the single-copy gene FISH, multi-round FISH with tandem repeats were hybridized to distinguish the homoeologous chromosome groups (Figure 4A–D, Figure S3). This showed that STlib_96 could detect the polymorphism in 5St_L, and STlib_117 signals were visible at the centromeric regions and both ends of chromosomes. To simplify the number of repeat probes used for chromosome identification, we used STlib_96 and STlib_98 as a mixture probe. Intense mixed STlib_96 + STlib_98 signals were mainly found at the subterminal chromosome regions, as well as at the centromeres of 4St and 6St. The summarized idiogram represents the cytogenetic karyotype of *P. libanotica* PI 228392 according to its homoeology with bread wheat, as determined using cDNA and generated repeats probes (Figure 5, Table 1 and Table 2). The homologous chromosome pairs 1St, 3St, 4St, and 6St were identical, whereas the 2St, 5St, and 7St homologous chromosome pairs displayed diversity which might indicate the heterozygosity of individual plants.

### 2.3. Intraspecies Polymorphism in P. libanotica Populations Based on Tandem Repeats and rDNA Probes

**Figure 6:**
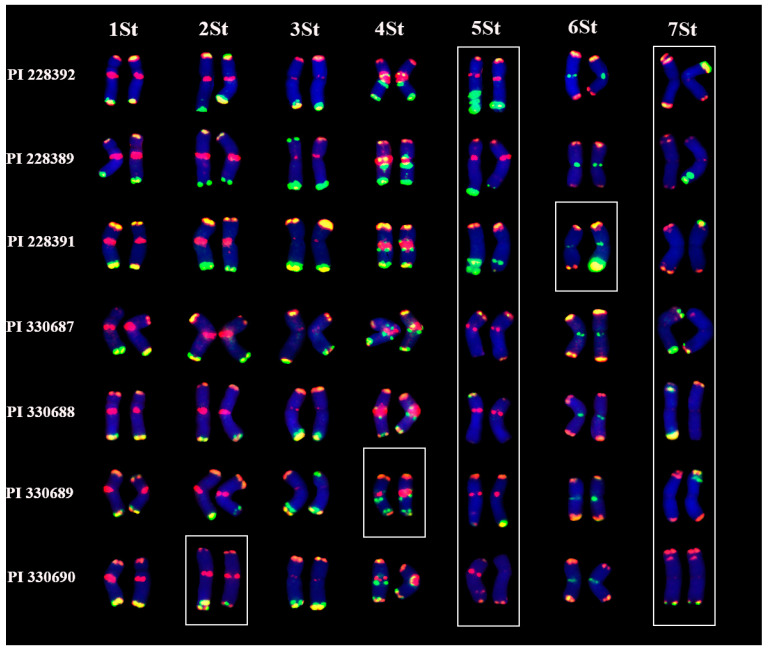
Chromosome polymorphism of seven *P. libanotica* accessions. The patterns of each chromosome pair were characterized by STlib_96 + Stlib_98 (green) and STlib_117 (red). The white box presents chromosome pairs with different signals.

The intraspecific genetic polymorphism in *P. libanotica* was determined in seven individual plants from different regions and was analyzed by FISH probes using STlib_96 + STlib_98, and STlib_117 (Figure S4). These accessions were arranged according to their signal similarities with the *P. libanotica* PI 228392 (Figure 6). All chromosomes from the analyzed accessions could be distinguished. We identified that 1St and 3St chromosome pairs were conserved across different accessions, whereas 2St, 4St, 5St, 6St, and 7St exhibited variations, particularly in 5St and 7St chromosome pairs (Figure 6). The abundant chromosome variations among accessions were in accordance with the homologous heterozygosity in *P. libanotica* PI 228392. 

**Figure 7:**
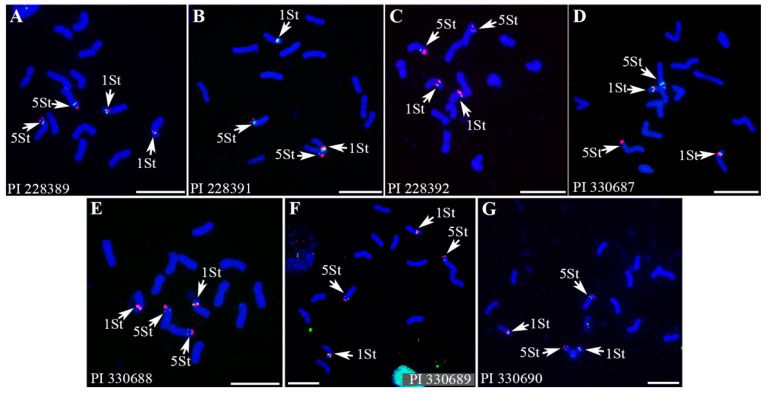
FISH on mitotic chromosomes of seven *P. libanotica* accessions with 45S rDNA (magenta) and 5S rDNA (green). (**A**) 45S rDNA (magenta) and 5S rDNA (green) in *P. libanotica* accession PI 228389. (**B**) 45S rDNA (magenta) and 5S rDNA (green) in *P. libanotica* accession PI 228391. (**C**) 45S rDNA (magenta) and 5S rDNA (green) in *P. libanotica* accession PI 228392. (**D**) 45S rDNA (magenta) and 5S rDNA (green) in *P. libanotica* accession PI 330687. (**E**) 45S rDNA (magenta) and 5S rDNA (green) in *P. libanotica* accession PI 330688. (**F**) 45S rDNA (magenta) and 5S rDNA (green) in *P. libanotica* accession PI 330689. (**G**) 45S rDNA (magenta) and 5S rDNA (green) in *P. libanotica* accession PI 330690. White arrows indicate chromosomes with secondary constriction regions. Scale bar equals 10 μm.

The 45S and 5S rDNA were investigated to identify rRNA encoding chromosomes in different *P. libanotica* accessions (Figure 7). Preserved 45S and 5S rDNA signals were observed on the short arms of 1St and 5St chromosome pairs with secondary constrictions. In the 1St chromosome, 45S and 5S rDNA were closely localized in the middle of the short arm, where the secondary constriction region could be observed. In the 5St chromosome, the 45S rDNA displayed a signal close to the end of the 5St_S, with 5S rDNA localized in the interstitial region.

## 3. Discussion

### 3.1. Characterization of the Cytogenetic Karyotype of P. libanotica Using Wheat cDNA Sequences

**Figure 8:**
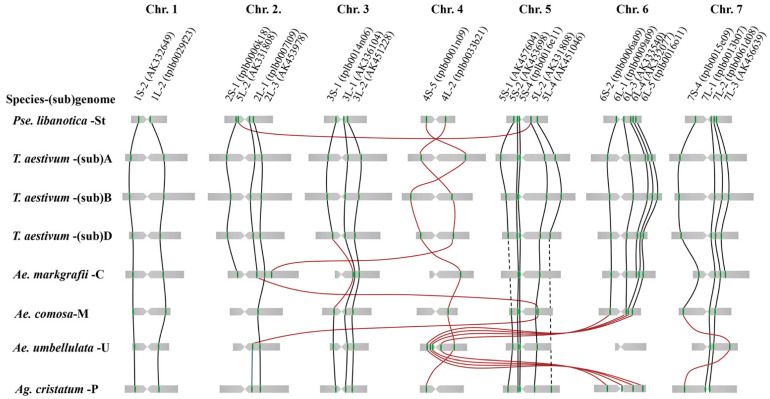
Cross-genome homoeologous within St genome of *P. libanotica*, A, B, and D sub-genome of *T. aestivum*, C genome of *Ae. markgarii*, M genome of *Ae. comosa*, U genome of *Ae. umbellulate*, and P genome of *Ag. cristatum.* Relatively chromosome length and gene position data of A, B, and D sub-genome of *T. aestivum* were prepared based on Chinese Spring reference genome (RefSeq v2.1) in the WheatOmics website (http://202.194.139.32/blast/blast.html, accessed on 2 November 2022) [35], C genome was generated from the publication of Danilova et al. [18], M, U, and P genomes were referred to by Said et al. [19,20]. The green dot on the chromosome indicates the cDNA sequence, and the red dot is a duplication of the original sequence. Black lines cross-genome indicate the collinearity and red lines present chromosome structure variation.

As summarized in Figure 8, the homeolog of the St genome from *P. libanotica* was compared with the A, B, and D subgenomes of common wheat, the C genome of *Ae. markgrafii*, the M and U genomes of *Ae. comosa* and *Ae. umbellulata* [19], and the P genome of *Ag. cristatum* [21] based on cDNA-FISH probes of Danilova et al. [18], respectively. The chromosome 4St in *P. libanotica* presented a major structural inversion compared with the A subgenome of the *T. aestivum*. This is a well-known 4A chromosome pericentric inversion during hexaploid wheat and tetraploid *T. turgidum* L. polyploidization [31,32]. A 5St_L fragment was duplicated and specifically inserted into the 2St_S of *P. libanotica,* which may indicate the evolutionary differentiation of the St genome from other Triticeae species. Previously, the karyotype in perennial Triticeae species was established based on their chromosome sizes [33]; however, the chromosome length and arm ratio would be affected by the pre-treatment method, cell cycle, and the measurement program. The single-copy gene FISH facilitated the identification of the homoeologous chromosome and improved the potential of the comparative cytogenetic karyotype [20,34]. These results will provide resources for studying genome organization and the evolution of the St genome and St-containing polyploid species.

### 3.2. Utilization of Three Generated Repeat Probes to Identify Individual St-Chromosome Pairs and Derived St Chromosome of P. libanotica

Cytogenetic and molecular markers have been used to qualitatively detect the St sub-genome in polyploids [16,25,26], however, individual St-chromosome pairs have been rarely identified in diploid *Pseudoroegneria* species. The newly developed FISH probe St_2_-80 in *P. libanotica* and transposable elements from *P. stipifolia* allowed the St sub-genome determination in St-containing allopolyploids [25,26]. Wang et al. [21] processed EST-SSR and 3468 genotyping-by sequencing (GBS) markers mapping population to identify the seven homoeologous groups of the St genome in the diploid *P. spicata*. Unfortunately, no cytogenetic marker was developed to distinguish different St-chromosome pairs. In this study, we generated three repeat probes, namely STlib_96, Stlib_98, and STlib_117, which could specifically identify seven individual St-chromosome pairs in the ancient St genome species-*P. libanotica*. 

Until now, no wheat-*P. libanotica* breeding materials have been generated. Nevertheless, St-containing species such as tetraploid *Elymus sibiricus* Linn. (StStHH genome) [36], *Roegneria ciliaris* (Trin.) Nevski (StStYY genome) [37], hexaploidy *Campeiostachys kamoji* Ohwi (StStYYHH genome) [38], *E. repens* (L.) Gould (StStStStHH genome) [39], and *Thinopyrum intermedium* (Host) Barkworth et Dewey (E^e^E^e^E^b^E^b^StSt genome) [40] have been extensively used for improving resistance to stripe rust and Fusarium head blight, as well as tolerance to drought and saline-alkali stresses in wheat. However, a shortage of cytogenetic and molecular markers, heterozygosity, and genetic diversity prevent the identification of homoeologous chromosomes in polyploids and hamper mapping resistance genes and uncovering their functional mechanisms. In this study, STlib_96 and STlib_98 displayed no signal in common wheat. Thus, they are promising due to their potential to identify *P. libanotica* derived St chromosomes or segments in the background of common wheat after wide hybridization. 

### 3.3. Secondary Constrictions and Nucleolus Organizing Regions (NORs) 

We verified that chromosome pairs with NOR were 1St and 5St in the St genome, but not the nominated As and Ds in diploid species *P. spicata* with the St genome [41], or 6St and 7St in the St sub-genome of hexaploidy *Kengyilia* species based on chromosome length [42]. Although in a majority of the Triticeae species, major NORs loci have been found on chromosomes 1 and 5, smaller or less active sites were also detected outside the NORs in different species [43]. There has been no information about the genomic distribution of 5S rDNA sites in *P. libanotica*. We located the 5S rDNA locus in the intercalary regions of the short arms of chromosomes 1St and 5St, and it is related to the 45S rDNA locus. In some Triticeae species, the 5S rDNA locus was found only in the homoeologous group 5 [20,44,45,46]. It was reported that the 5S sequences comprised a higher diversity than sequences of the 45S rDNA internal transcribed spacer (ITS) region [47]. The presence of additional sites in various organismal groups has been explained as a possible remnant of gene duplication [48], and these sites fit well within the range of nucleotide diversities found in the Triticeae [49,50].

### 3.4. Intraspecific Polymorphism in Diploid Pseudoroegneria Species with St Genomes May Be Accounted for by Open-Pollination and Climate during the Long Evolution Time

Alterations in the number and distribution of tandem repeats are a crucial manifestation of intraspecific genetic variation [23,25,26,41,42]. In this study, three generated repeat probes—STlib_96, STlib_98, and STlib_117—were developed to construct the cytogenetic karyotype of *P. libanotica* PI 228392. However, this is not sufficiently representative, because the 5St and 7St displayed abundant diversity within homologous chromosome pairs and across accessions. Heteromorphism in homologous chromosomes manifests the genetic variations, possibly resulting from altered chromosome structures, such as chromosome rearrangements, translocations, and inversion between chromosomes within these accessions [20,50,51,52,53]. Moreover, heteromorphic chromosomes could frequently occur from open pollination plants [18,54,55]. This phenomenon has been reported in Triticeae species such as *Ae. speltoides* Tausch. [55], *Secale cereale* L. [56], and different genotypes of perennial *Ag. cristatum* [20,33]. In the St-containing allotetraploid *R. ciliaris* [57] and allohexaploid *Kengyilia* spp. [32], the St sub-genome also displayed higher variations, especially the 7St. Thus, we speculate that high-level genetic heterogeneity in 5St and 7St of diploid parents not only spurs the establishment of fitness and abundant genetic diversity but also contributes to a the genetic advantage in terms of the richness of their polyploid offspring species in Triticeae. Many natural hybrids between various St-containing polyploid species have been reported [58,59,60,61], further enlarging the richness of St-containing descendants.

## 4. Materials and Methods

### 4.1. Materials 

Materials used in this study are listed in Table 4. The seeds of the *Pseudoroegneria* species were provided by the United States National Plant Germplasm System (Pullman, WA, USA). Wheat cv. Chinese Spring was collected by the researchers of the Triticeae Research Institute (Sichuan Agricultural University, Chengdu, China). The voucher specimens of all genotypes were deposited in the Herbarium of Triticeae Research Institute, Sichuan Agricultural University, China. Plants were grown under greenhouse conditions: 16 h light/8 h dark and temperatures of 20–24 °C day/17–19 °C night. 

### 4.2. Generation of Single-Copy Gene Probes 

We selected 24 cDNA sequences previously mapped to the seven homoeologous chromosomes of bread wheat by [18] (Table S1). Total leaf RNA was extracted from wheat using an RNA isolation kit (TIANGEN, cat. DP432, Beijing, China). RNA integrity was assessed using the RNA Nano 6000 Assay Kit of the Bioanalyzer 2100 system (Agilent Technologies, Santa Clara, CA, USA). Each single-copy sequence was amplified in 50 μL of the reaction mixture, containing 25 μL of Taq Master Mix (Vazyme, cat. P112-AA, Nanjing, China), 2.5 μL of each primer (10 mM), 200 ng of template cDNA, and dH_2_O was added to the final volume. Each amplified fragment was cloned into the PMD19-T vector according to the manufacturer’s instructions (Takara, cat. 6103, Dalian, China), verified by sequencing (Sangon Biotech, Shanghai, China), and blasted against the Chinese Spring reference genome (RefSeq v2.1) on the WheatOmics website (http://202.194.139.32/blast/blast.html, accessed on 2 November 2022) [35] to confirm the copy number. The sequence length varied from 1166 bp to 4155 bp. 

### 4.3. Identification of St Genome Tandem Repeats Using Similarity-Based Read Clustering and Probes Labeling

Genomic DNA of *Pseudorogneria* was isolated from leaves with DNeasy plant mini kit (Qiagen, cat. 69104, Dusseldorf, Germany). For Illumina (San Diego, CA, USA) short-read sequencing, libraries were size-selected for PE150 sequencing. Sequencing libraries with insert sizes of 350 bp were constructed and sequenced using an Illumina HiSeq X Ten platform at the Novogene Bioinformatics Institute, Beijing. Next-generation sequencing was conducted by Novogene (Beijing, China) (paired-end, 2 × 101 cycles, Illumina Hiseq-PE150 platform). In total, ~2.0 Gb of raw data were obtained from the St genome corresponding to 0.5× genome coverage. All data supporting the findings of this study are available in this paper. Raw sequence data have been deposited at the National Center for Biotechnology Information under BioProject accession No. PRJNA843189.

In silico identification of St genome-enriched candidate repeats was performed by similarity-based clustering of Illumina reads in the RepeatExplorer pipeline (https://galaxy-elixir.cerit-sc.cz, accessed on 20 November 2022) [30,62]. Based on TAREAN, satellite repeats, LTR-retrotransposons, 45S and 5S rDNA, and the remaining clusters with default size threshold were identified. All putative clusters represent at least 0.01% of the input reads, and the monomer lengths and consensus sequences of newly identified satellite repeats were also determined [28]. Putative repeats were compared with the database from Repbase (https://www.girinst.org/repbase/, accessed on 20 November 2022) and National Center for Biotechnology Information (NCBI) by BLAST (program selection: megablast, https://blast.ncbi.nlm.nih.gov/, accessed on 20 November 2022). Primers were designed using the program Primer3Plus-PickPrimers (http://www.primer3plus.com, Table S1). Genomic DNAs were applied as templates, and PCR products were cloned into the PMD19-T vector according to the manufacturer’s instructions (Takara, cat. 6103, Dalian, China) and verified by sequencing by Sangon Biological Engineering and Technology Service Ltd. (Shanghai, China). Tandem repeats and cDNA products were purified and directly labeled by nick translation with the dUTP-ATTO-550 or dUTP-ATTO-488 labeling kit (Jena Bioscience, cat. PP-305L-550, cat. PP-305L-448, Jena, Germany).

### 4.4. Preparation of Chromosome Spreads

Rapidly growing roots were collected from seedlings or adult plants. Roots were treated with nitrous oxide (N_2_O) at 0.1 MPa for 2 h and then fixed with 90% (*v*/*v*) glacial acetic for 5 min. Afterward, the roots were washed twice with distilled water. Meristems were digested with 4% cellulase onozuka R-10 (Yakult, Tokyo, Japan) and 2% pectolyasein Y-23 (Yakult, Tokyo, Japan) in 0.01 M citrate buffer for 60 min at 37 °C. The preparation of mitotic chromosome spreads was performed according to Aliyeva-Schnorr et al. [63] with minor modifications. Briefly, after enzymatic treatment, meristems were washed twice in 75% (*v*/*v*) ethanol. Ethanol was replaced with 90% (*v*/*v*) acetic acid (10–15 μL per root meristem), and meristems were disintegrated using a plastic pistol. The obtained suspension was dropped onto slides under 50–55% humidity conditions. After drying, slides were checked by phase-contrast microscopy and dryly stored at −20 °C until used for FISH. 

### 4.5. FISH and Microscopy

Hybridization of single-copy gene probes was conducted according to Aliyeva-Schnorr et al. [63] with minor modifications. Briefly, hybridization mixture per slide (total volume = 20 μL), consisting of 50% *v*/*v* formamide, 2 × SSC, 0.1 M Tris-HCl pH 8.0, 0.05 M EDTA, 1 μg/μL salmon sperm DNA, and 300 ng cDNA probe/50 ng tandem repeat probe, was applied and denatured at 80 °C for 2 min on a hot plate. Slides were incubated overnight in a moister chamber at 37 °C. Afterward, coverslips were removed, and slides were washed at 55 °C 2× SSC for 20 min. Slides were treated with 2× SSC for 2 min at RT, dehydrated, and counterstained with 4′,6-diamidino-2-phenylindole (DAPI) in Vectashield. For multiple rounds of repeat cluster FISH, coverslips were removed, and stripping was performed as follows: slides with specimen were boiled in 2× SSC for 10 min, dehydrated for 10 min in each ethanol series (75%, 80%, and 95%), air-dried, and then slides were exposed to intense sunlight for at least 24 h. The hybridization of repeat cluster FISH probes was the same as the single-copy probes above. All hybridization signals were captured under the fluorescence Olympus BX63 microscope equipped with a DP80 CCD camera (Olympus, Tokyo, Japan). All images were collected in greyscale and pseudo-colored with Adobe Photoshop CS (Adobe). 

### 4.6. Chromosome Measurements

The ten best metaphase spreads were used to establish the idiogram. The chromosome ordering in the karyogram referred to seven homoeologous chromosome groups as common wheat according to the single-copy gene FISH [17]. The length of the single chromosome (T), the length of the long arm (L), short arm (S), satellite, and the position of the hybridization signal from the end of the long arm were measured with Image J (Image Processing, http://imagej.net) in micrometers (μm). The arm ratio was calculated by dividing the length of the longer arm of the chromosome by the length of the shorter arm (excluding the satellite length, arm ratio = L/S). The characteristic chromosome type determination is based on Levan et al. [64]. The relative length was determined by dividing the length of a particular chromosome (T) by the total length (H) of chromosomes in the haploid set (Relative length = 100 × T/H). The centromeric index was calculated by dividing the length of the shorter of the two chromosome arms by the length of the whole chromosome and expressed in percent (centromeric index = 100 × S/T). Individual chromosomes were classified according to the centromeric index. For each single-copy FISH signal site determination, ten different metaphase chromosomes showing a hybridization signal were measured. The relative cytological position (CP) was determined by dividing the distance from the end of the short arm to the signal site (SS) by the total length of the chromosome (CP = 100 × SS/T) [63].

## 5. Conclusions

We identified seven homoeologous chromosome pairs using 24 wheat single-copy gene probes and newly developed tandem repeat clusters, STlib_96, STlib_98, and STlib_117, from *P. libanotica* for rapid chromosome pair differentiation. Moreover, we assessed the intraspecific genetic polymorphism of *P. libanotica*. For the first time, we established the cytogenetic karyotype of diploid *P. libanotica*. The intraspecific results illustrate that the 2St, 5St, and 7St chromosome pairs display homologous heterozygosity, as well as vary among individual plants of *P. libanotica* plants. The results advance our understanding of the polymorphism among the *P. libanotica* population from the cytogenetic perspective and provide insights into the genome organization and evolution of the St genome and St-containing polyploids.

## Figures and Tables

**Table 1:** Relative position of cDNA probes on *P. libanotica* chromosome.

Probe	Chromosome*	GenBank No.	CP* (%)
1S-2	1St_S	AK332649	22.02
1L-2	1St_L	AK449552	46.52
2S-1	2St_S	AK454726	28.36
2L-1	2St_L	AK455013	52.26
2L-3	2St_L	AK453978	59.89
3S-1	3St_S	tplb0014n06	30.13
3L-1	3St_L	AK336104	46.52
3L-2	3St_L	AK451228	54.90
4S-5	4St_S	AK453437	18.62
4L-2	4St_L	AK449944	71.27
5S-1	5St_S	AK457604	32.99
5S-2	5St_S	AK453698	27.33
5S-4	5St_S	tplb0016e11	19.70
5L-2	5St_L	AK331808	59.42
5L-4	5L-4	AK451046	75.66
6S-2	6St_S	tplb0006a09	32.08
6L-1	6St_L	AK455396	53.76
6L-3	6St_L	AK333540	72.70
6L-4	6St_L	AK332077	75.43
6L-5	6St_L	AK458456	84.80
7S-4	7St_S	AK457210	23.24
7L-1	7St_L	tplb0013b07	55.99
7L-2	7St_L	AK453006	61.85
7L-3	7St_L	AK456639	65.47

Chromosome*: S and L indicated the short arm and the long arm of chromosome, respectively. CP* represented the relative cytological position, which was determined by dividing the distance from the end of the short arm to the signal site (SS) by the total length of the chromosome (CP = 100 × SS/T).

**Table 2:** Karyotype information in *P. libanotica*.

Chr.	Chr. Size (μm)				Arm Ratio (L/S)	Relative Length (T/H) × 100	Centromere Index (S/T) × 100	Chr. Morphology
	Total (T)	Short (S)	Long (L)	SAT.				
1St	4.61 ± 0.10	0.85 ± 0.03	2.55 ± 0.05	1.17 ± 0.03	2.96	13.30	44.67	sm + sat
2St	5.59 ± 0.15	2.37 ± 0.06	3.21 ± 0.10		1.36	16.13	42.41	m
3St	5.19 ± 0.12	2.15 ± 0.05	3.05 ± 0.07		1.42	14.70	41.37	m
4St	4.35 ± 0.10	1.87 ± 0.04	2.48 ± 0.07		1.33	12.20	42.99	m
5St	5.10 ± 0.12	1.52 ± 0.04	3.19 ± 0.09	0.41 ± 0.03	2.13	14.93	37.33	sm + sat
6St	4.56 ± 0.11	2.06 ± 0.06	2.51 ± 0.07		1.22	13.12	45.07	m
7St	5.44 ± 0.12	2.59 ± 0.06	2.85 ± 0.07		1.10	15.62	47.64	m
	34.78 (H)							

T indicated the total length of single chromosome. SAT. indicated the satellite chromosome, we represent the size of the secondary constriction region. H represented the total length of haploid chromosomes; m and sm indicated the middle centromere chromosomes and sub-middle centromere chromosome according to the arm ratio.

**Table 3:** Repeat composition of *P. libanotica* identified from clustering analysis of Illumina reads.

Repeat Type	Lineage/Class	Genome Proportion (%)
LTR retroelements Ty1_copia		8.14
	Angela	5.68
	SIRE	2.29
	TAR	0.17
Ty3_gypsy		14.50
	Athila	5.34
	TatV	2.81
	CRM	0.48
	Tekay	5.87
Other	EnSpm_CACTA	3.38
	MuDR_Mutator	0.01
	rDNA	0.34
	satellite	2.66
Unclaissified_repeat		9.74
Non-annotated sequences		15.90

**Table 4:** Materials in this study.

Materials	Accession	Polyploidy	Geographical Distribution
*P. libanotica*	PI 228389	2x = 14	East of Sanandaj, Kurdistan
*P. libanotica*	PI 228391	2x = 14	West of Ardabil, Iran
*P. libanotica*	PI 228392	2x = 14	Northeast of Kuhe Savalan, Azerbaijan
*P. libanotica*	PI 330687	2x = 14	Kandavan Pass, Iran
*P. libanotica*	PI 330688	2x = 14	Sirak-Sar, Armenia
*P. libanotica*	PI 330689	2x = 14	Sirak-Sar, Armenia
*P. libanotica*	PI 330690	2x = 14	Sirak-Sar, Armenia
*T. aestivum cv.* Chinese Spring		2x = 42	Sichuan, China

## Data Availability

The data presented in this study are available in Supplementary Materials.

## Supplementary Materials

The supporting information can be downloaded at: https://www.mdpi.com/article/10.3390/ijms232314818/s1.
