# Supplementary material for: Pseudorogneria libanotica Intraspecific Genetic Polymorphism Revealed by Fluorescence In Situ Hybridization with Newly Identified Tandem Repeats and Wheat Single-Copy Gene Probes
Source: Int J Mol Sci. 2022 Nov 26;23(23):14818. doi: 10.3390/ijms232314818 (PMC9737853; doi:10.3390/ijms232314818)
Supplement: Supplementary file 1 [file ijms-23-14818-s001.zip › Supplementary materials.pdf]

Supplementary materials

Supplementary Table S1 Probe information used in this study

**Probe name**	**Primer name**	**Primer sequence (5'-3')**	**Fragment size (bp)**	**GenBank no.***	**Reference**
STlib_96	STlib_96_F	CGTTTTGGCAGGTTGTGGAG	570 bp	*OL685355*	This study
	STlib_96_R	GCTTCGGCAATGTTTCGGAG			
STlib_98	STlib_98_F	GTCACGGCTTGGACACAAAC	503 bp	*OL685354*	This study
	STlib_98_R	AGGGCATTAACTCGCACACA			
STlib_117	STlib_117_F	GGAGGCTCTTTGTTGCATCG	554 bp	*OL685353*	This study
	STlib_117_R	CGGACGAGTGGGATATGGAC			
1S-2	1S-2_F	CAAAAGGTCGTCACGGAGGA	2438 bp	AK332649	Danilova et al., 2017
	1S-2_R	TTCTGCTGTCACATGACCCC			
1L-2	1L-2_F	ACGCCATGGTTGTCTCGATT	2596 bp	AK449552	Danilova et al., 2017
	1L-2_R	AGCCGGCTTGTTCCTAACTC			
2S-1	2S-1_F	AAGGTCTGCGACGCCATATC	3018 bp	AK454726	Danilova et al., 2017
	2S-1_R	CAACCTTGCAGCCACAGAAC			
2L-1	2L-1_F	GTCCTTGGGTTATTCGGCCA	2130 bp	AK455013	Danilova et al., 2017
	2L-1_R	CAAACACTCGAAGCTTGCCC			
2L-3	2L-3_F	TGCCAGTGACCGGTTGATAC	2615bp	AK453978	Danilova et al., 2017
	2L-3_R	AGCTGCACATGTCATGGTGA			
3S-1	3S-1_F	CATCTCCCACCAGCCACATT	2668 bp	tplb0014n06	Danilova et al., 2017
	3S-1_R	AATTGCTGGGAGGGGCTAAC			
3L-1	3L-1_F	GTCTCTCTCGCTGAATCCGG	3126 bp	AK336104	Danilova et al., 2017
	3L-1_R	TGCGACTATCGTAGCAGCAG			
3L-2	3L-2_F	CGGAGCACTCTCACCAACTT	2711bp	AK451228	Danilova et al., 2017
	3L-2_R	CTCGCGGTTTTGTGTTCGTT			
4S-5	4S-5_F	GATTCATTCCGCGCAACCTC	1166 bp	AK453437	Danilova et al., 2017
	4S-5_R	ATGGCTCGTCGAGATGTTCC			
4L-2	4L-2_F	GGTGAAGGCGTCAAGGAAGA	2730 bp	AK449944	Danilova et al., 2017
	4L-2_R	GCTTGGGGATTTCTCCAGCT			
5S-1	5S-1_F	GAGACAACTTTTGCAGGCCG	2505bp	AK457604	Danilova et al., 2017
	5S-1_R	GAACTTCTCCGTCACCTGCA			
5S-2	5S-2_F	TCCGTTGAGCGACCAATACC	2537bp	AK453698	Danilova et al., 2017
	5S-2_R	TGTGCCCCGATCTTCGAAAA			
5S-4	5S-4_F	GAGTGGGGGATCCTCTTCCT	2223 bp	tplb0016e11	Danilova et al., 2017
	5S-4_R	CTTGCACAAATCACCCTGCC			
5L-2	5L-2_F	TGCTGACGCTGGTGATGATT	3856 bp	AK331808	Danilova et al., 2017
	5L-2_R	AGACACCTCCACCTCCCTAC			
5L-4	5L-4_F	CGCGCTACTACGAGATCCTG	1245bp	AK451046	Danilova et al., 2017
	5L-4_R	ACAACATCCTGCCTGCCTAC			
6S-2	6S-2_F	CGGTGATATGTCCGTGGGAG	1875 bp	tplb0006a09	Danilova et al., 2017

	6S-2_R	CAGGACTGGCTCAAGCTCAA			
6L-1	6L-1_F	CCTCTACTGCTCTCATGCCG	2942bp	AK455396	Danilova et al., 2017
	6L-1_R	GTTCATGGCCATCGATGTGC			
6L-3	6L-3_F	GGGCAGAACATTGCATGCAA	3072 bp	AK333540	Danilova et al., 2017
	6L-3_R	TTGGGGCCAGAATAGCTTGG			
6L-4	6L-4_F	GCTCTATGACGATGCCGACA	4155 bp	AK332077	Danilova et al., 2017
	6L-4_R	CAACATCTAACATGCGCCGG			
6L-5	6L-5_F	GAGCGAGTAGGGTTGTTGCT	2155 bp	AK458456	Danilova et al., 2017
	6L-5_R	CTCCCCGTGATCAGATGCTC			
7S-4	7S-4_F	TGGCGGTGTAATCGAGATCG	3068 bp	AK457210	Danilova et al., 2017
	7S-4_R	TTCTCTTGTCGAGGCAGCAG			
7L-1	7L-1_F	TATTCCATCGGGCATCCTGC	2484bp	tplb0013b07	Danilova et al., 2017
	7L-1_R	ACAGCACGCTGATGGTTACA			
7L-2	7L-2_F	TGCAGACAGCTACCTGGTTG	2621 bp	AK453006	Danilova et al., 2017
	7L-2_R	GCTAAATCATGTGCCCGCAG			
7L-3	7L-3_F	CTTCCGTCCCATCCAAAGCT	3334bp	AK456639	Danilova et al., 2017
	7L-3_R	TACAACAACCGAGGAACCCG			

GenBank no.* with Italic were submitted by authors in this study.

Supplementary data

>STlib_96
ACCGGGGTATACGAATTTTCGTTGCGGTCGTTTTGGCAGGTTGTGGAGCTCAAACGGAGT
TCGTATGACCAGATTACGCCCGTTCTACGGATACAACCTAAATGGATTTCAGCACCGGGG
CAGACCGTGAGTCGAGCTTCCAAGGTAAGTTGATCGCGCCGGGGAGCCATCTTTCACCA
CTCGGAATGACCTATTATTTTGCGTGTAAATGATAAGAGCGCAGAAGAGCTGCTATGCAC
ATTTTCGTCCAAGAACGGCGCATGTTCTTGCCCAAACATGCGGCTGGCAGCGGGATGGGT
GATAGAGCATGCAAAACTTCATTTTTGGGGGTTTTGGTGCACCAAGGAGCCTCCTCCTTC
TATCCAGACTGACATGTAGGCCTTGTTGGTGCACAAATGGAATCATATCTCATTATTCTGTA
TGTTATTGTCCCAACATGTGTCCATGGGTGAGCTCACGTAACGATGGTAGAGGCTAGGAG
TGTACACGCCAGCGATTAAATCATCAGGAACTCAATTTTGCAAGCCGGTTTTGGCCTCCG
AAACATTGCCGAAGCCGACGAAGTGGGCC

>STlib_98
ATTGGTGTCACGGCTTGGACACAAACATTGAGAACCATGGCTTTTAAATTCCAAAAAATT
CAAAAATGATCAGAAATACATGAAACCTTGCTTGATGTCATGAAATGGCACCAAGATGCT
GTGGTAAAAGAATTGGCCACTTTGACGAAAGTTTTGACACACACCTCTCACAAATCGGA
GCATCTCACGAGAAGACTCGTGGTTCCGAGAGGGAACAATGCATGTTTGATGACGAACC
GGCGCTCGCTTCCTCGTATGGCCTTGAATTTTTTTCTACCGTTAACATGCACTAATACAACT
GTCATGTTAAAATTTGGAAATTTTCAGGGTTCATTTGACCTTTTTAAGACATTAAGTGAGT
TTCTAGCCATATAATGGCCGTAATTCAAATTTGAACTACATGTACATGCAACAGCAAACCA

TCATGGGTTGGAAAATCATATTTGTGTCCTTGTGTGCGAGTTAATGCCCTGTGCAGTAAAT
TGGAAGGAATTTTCAAACAT

>STlib_117

ACGAACCAGAAAGACTACAGGTTCAGTCTGGACAGAAGGAGGAGGCTCTTTGTTGCATC
GAAACCCAAGAATTCATGCTTCACATGTTCTATCACCCATCCCGCTACCACCCTCACTTTT
TCACCAGCAGATGCATTATTCTTGGATGATAAAGTACGTAACAGCTCATCCTCATTATGTTT
ATGCACACGAAAAATTATAGGTCATTCCGAATGGTGCAAGATGGCTCGTCGATGCGATCA
AGTTACCCTGAAAGTTGTATTCACGGTCTGTCCCCCGATGTTGTATCCATAAAACGGCCAC
AATTTGGCCATACAAACTCCGTTGGAGCTCCATGACCCGCCAAATCGATCACAACGAAAA
GTTGTATGCGTCCATATCCCACTCGTCCGCTTCGACAACATTTCGGAGGCCAGATCCGGCT
TGTAAAATTGAGTTTCGGATGATTTTATCTTTGGCGTGTACTCTGCCACCCTCTACCACCCT
TCCGTGAGCTCACCCACGGACAGATATATGGGCAATAACGTATGGAACAATACACCATCC
TTCCATTC
