# Supplementary figures and images for: Pseudorogneria libanotica Intraspecific Genetic Polymorphism Revealed by Fluorescence In Situ Hybridization with Newly Identified Tandem Repeats and Wheat Single-Copy Gene Probes
Source: Int J Mol Sci. 2022 Nov 26;23(23):14818. doi: 10.3390/ijms232314818 (PMC9737853; doi:10.3390/ijms232314818)

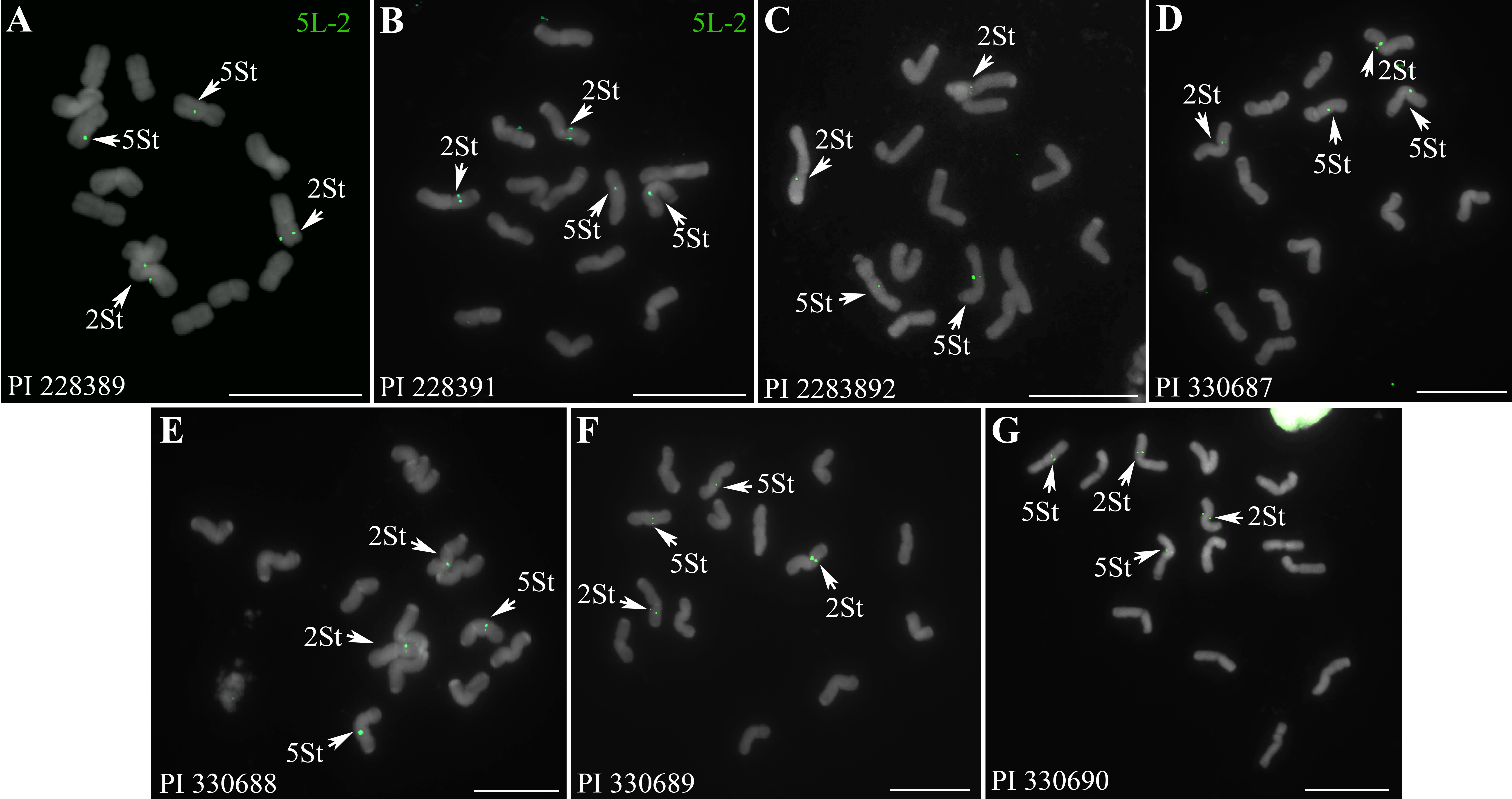

Supplement: Supplementary file 1 [file ijms-23-14818-s001.zip › Figure S1.tif]

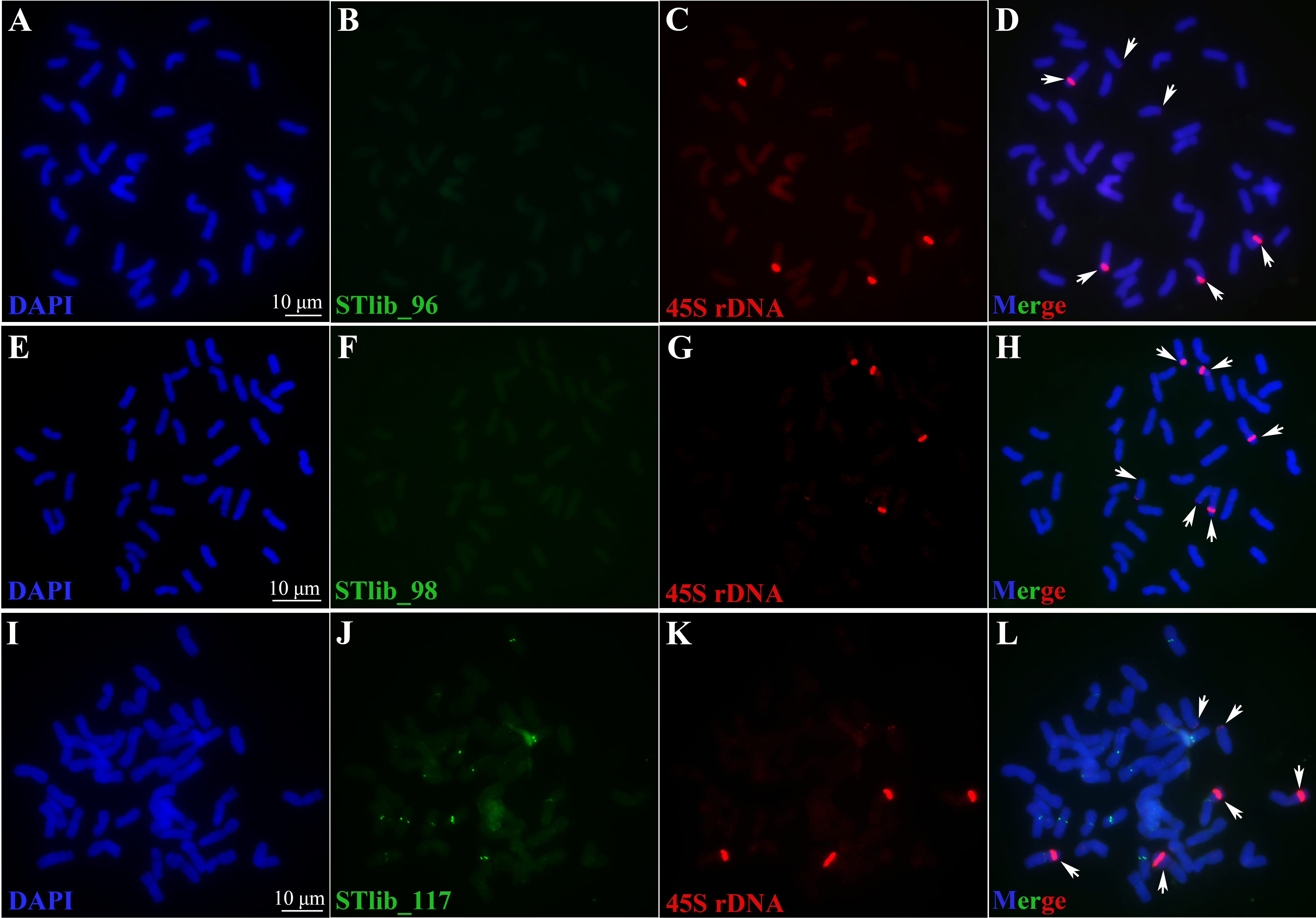

Supplement: Supplementary file 1 [file ijms-23-14818-s001.zip › Figure S2.tif]

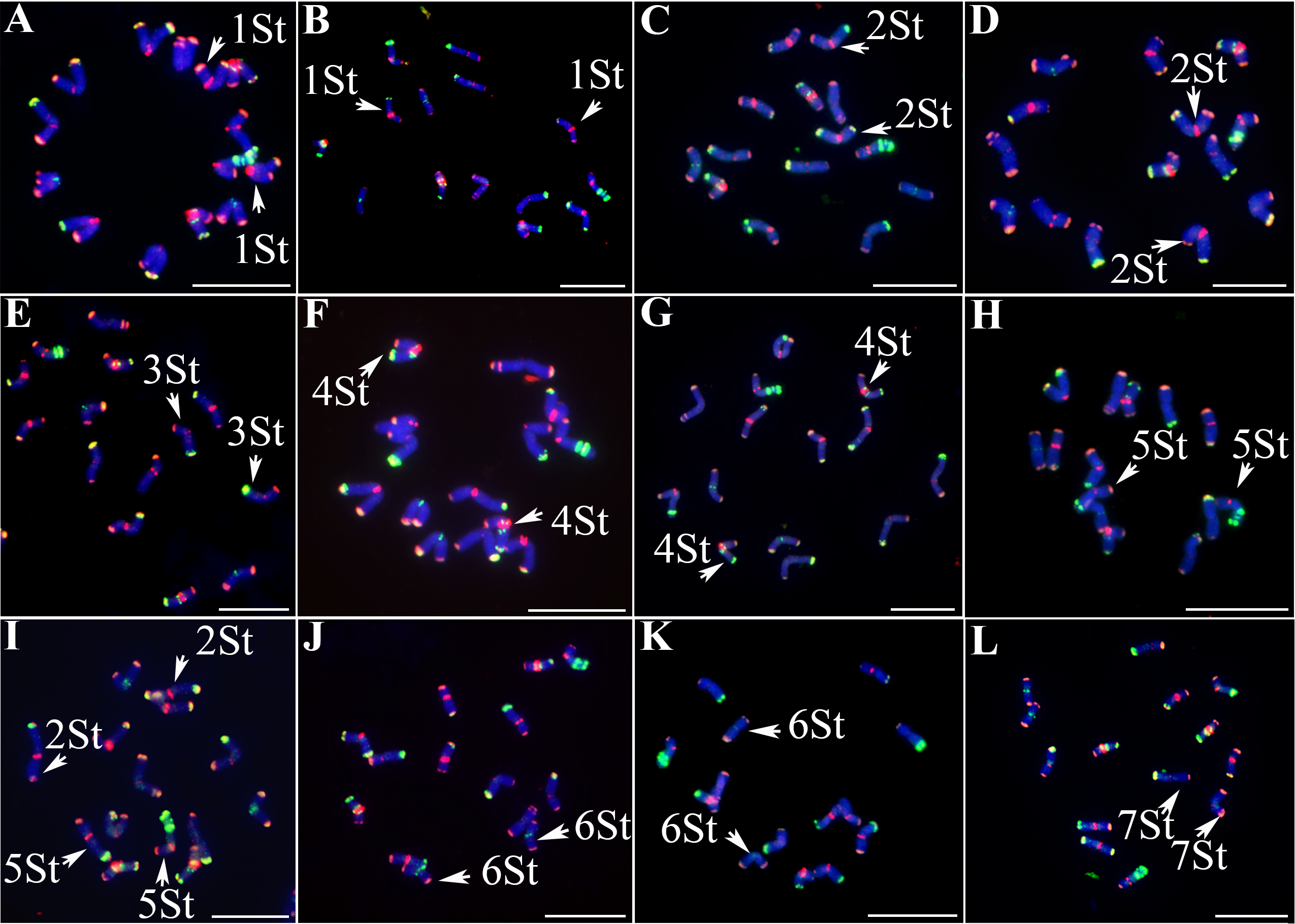

Supplement: Supplementary file 1 [file ijms-23-14818-s001.zip › Figure S3.tif]

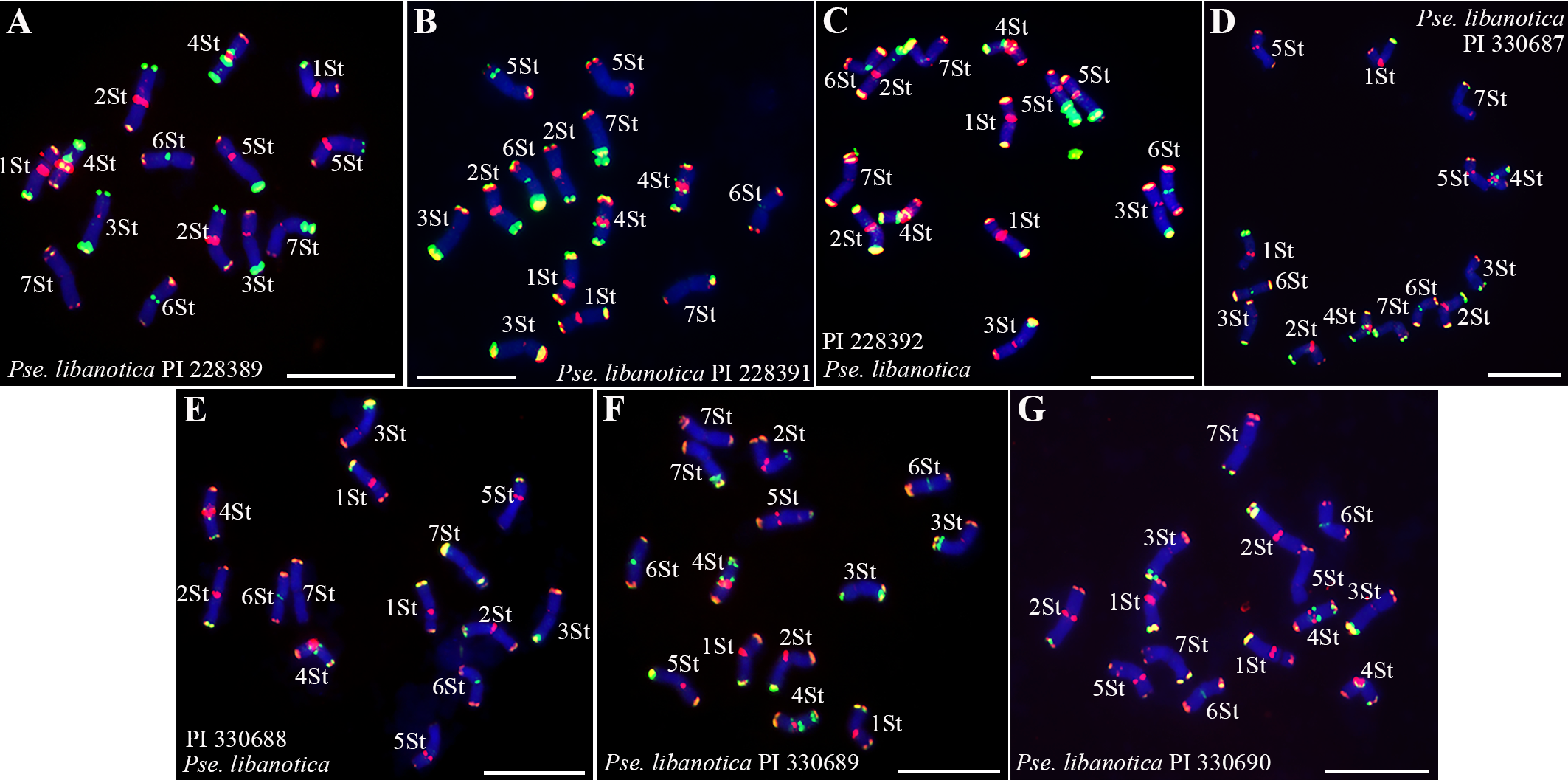

Supplement: Supplementary file 1 [file ijms-23-14818-s001.zip › Figure S4.tif]
